# The relationships between resilience and student personal factors in an undergraduate medical program

**DOI:** 10.1186/s12909-021-02547-5

**Published:** 2021-02-18

**Authors:** Ardi Findyartini, Nadia Greviana, Azis Muhammad Putera, Reynardi Larope Sutanto, Vernonia Yora Saki, Estivana Felaza

**Affiliations:** 1 Medical Education Center, Indonesia Medical Education and Research Institute (IMERI), Faculty of Medicine Universitas Indonesia, Jakarta, Indonesia; 2 Department of Medical Education, Faculty of Medicine Universitas Indonesia, Jakarta, Indonesia; 3 Undergraduate Medical Program, Faculty of Medicine Universitas Indonesia, Jakarta, Indonesia

**Keywords:** Resilience, Undergraduate, Medical students, Coping mechanism, Personality traits

## Abstract

**Background:**

Resilience is an essential aspect of wellbeing that plays a major role in undergraduate medical education. Various personal and social factors are known to affect resilience. Empirical evidence remains limited regarding resilience and the personal factors that affect it among undergraduate medical students in an Asian setting. Therefore, this study aims to identify undergraduate medical students’ level of resilience and its relationships to personal factors in Indonesia.

**Methods:**

This cross-sectional study was conducted among undergraduate medical students in years 1–6. Respondents were asked to complete three validated questionnaires: the Connor-Davidson Resilience Scale (CD-RISC) to measure resilience, the Brief-COPE to assess coping mechanisms, and the Big Five Personality Test to measure five personality dimensions. Descriptive and Pearson’s correlation analyses were completed to explore relationships between each variable. Regression analysis was completed to analyze the extent to which coping mechanisms, personality, and academic achievement explained the variation in resilience scores.

**Results:**

A total of 1040 respondents completed the questionnaires (a 75.42% response rate). Students in both preclinical and clinical stages had quite good levels of resilience and higher scores on adaptive coping mechanisms than on maladaptive coping mechanisms. Adaptive and maladaptive coping mechanisms, Big Five Personality traits (extraversion, agreeableness, conscientiousness, neuroticism, and openness), and students’ academic achievement explained 46.9% of students’ resilience scores.

**Conclusions:**

Although the resilience scores in this study were comparable to resilience scores among undergraduate medical students in other settings, we found that coping mechanisms, personality traits, and academic performance may predict resilience among medical students.

## Background

Resilience has been recognized as an essential aspect of wellbeing in medical education that enables students to recover from adversities and challenges [1, 2]. The concept of resilience can be seen from psychological, sociological, ethical, and moral perspectives. Thus, resilience has been defined as “a dynamic capability which allows people to thrive on challenges given appropriate social and personal contexts” [2]. Despite the dynamic nature of resilience and its multidimensional factors [3, 4], it is believed that resilience can be learned [3]. More importantly, resilience can also be promoted among individuals, resulting in better psychological and physical states [4].

Resilience plays a major role among undergraduate medical students due to their heavy workloads, which are associated with curricula, transitions in medical training, and changes in their personal lives. Compared to other student groups of the same age, medical students are more likely to endure negative psychological states, such as depression, burnout, psychosomatic complaints, decreased empathy, thoughts about quitting school, self-harm, and suicidal ideation, as well as poor academic performance [5, 6]. One study found that 27.2% of medical students were depressed or showed depressive symptoms, while 11.1% of medical students showed suicidal ideation [7]. Therefore, an emphasis on cultivating resilience is critical for medical students to mitigate and cope with the negative effects of their stressors [3, 8].

Various factors may affect the development of resilience among medical students. Social support and coping styles are two of these factors [3]. A cross-sectional study conducted in Brazil found that older students, those with a good perception of health, and students refusing to use habit-forming medications all demonstrated increased resilience [5]. Personality type is also suggested as a factor in resilience. Based on the Big Five Personality framework, personality traits that support better resilience are openness, agreeableness, conscientiousness, and extraversion due to better social support, adaptive coping, and positive emotions. On the other hand, neuroticism, which is related to negative emotions and maladaptive coping, tends to reduce resilience [9, 10]. Academic performance reportedly correlates with students’ resilience via academic satisfaction [11]. Another study also suggested that students’ past performance can predict future course performance for high- and low-achieving students. While self-assuredness is an individual factor that may improve performance among low achievers, drive is a predictor of performance among high achievers [12].

Current medical students from Generation Z are known to be more prone to insecurities, depression, and anxiety. Moreover, their overreliance on adults and information technology during their formative years may also affect their ability to cope with challenges in life [13]. It should be realized, however, that different societies have different values and norms that must be considered to understand the factors that influence resilience in the current generation of medical students [14]. Studies on medical students’ resilience in Asian settings are limited [14–16]. Few studies have examined personal factors and their relationships to resilience among undergraduate medical students in settings where cultures and conditions are different from the West [17, 18]. Therefore, this study aims to identify the relationships between resilience and personal factors (personality, coping mechanisms, and academic performance) among undergraduate medical students in Indonesia. The research questions are as follows: a. What is the resilience level of undergraduate medical students from different years of study? b. How do medical students’ personality, coping mechanisms, and academic performance affect their resilience? This study helps inform medical education in this setting to better support resilience throughout their medical training by considering individual factors.

## Methods

### Context

The undergraduate program of the Faculty of Medicine Universitas Indonesia (FMUI) uses a 5.5 year-competency-based curriculum. The medical school accepts high school graduates, who are required to take preclinical courses during the first 3.5 years (7 semesters). In the preclinical stage, the curriculum is developed progressively with a system-based learning approach. The primary learning activities during this stage include problem-based learning, collaborative learning, interactive lectures, and laboratory and clinical skills sessions, with sufficient time for independent learning.

The clinical stage begins in the eighth semester with a clinical rotations approach. Students are assigned to lengthy clinical rotations in internal medicine, surgery, obstetrics/gynecology, and pediatrics during their first year in the clinical stage, followed by shorter clinical rotations during the second year of their clinical stage. Following the short clinical rotations, students are placed in primary care centers and district hospitals for a pre-internship program. The primary learning activities during the clinical stage are conducted in the workplace, where students are exposed to patient encounters under supervision, case presentations, and clinical skills sessions.

### Study design

We utilized a cross-sectional design with a total sampling approach. Students in years 1–6 in preclinical and clinical stages were invited to complete questionnaires from December 2019 to January 2020. This study was approved by the Research Ethical Committee of FMUI and Dr. Cipto Mangunkusumo (National Hospital No. KET-1299/UN2.F1/ETIK/PPM.00.02/2019).

### Population and respondents

Respondents in this study were undergraduate medical students at FMUI in preclinical and clinical stages with active enrolment status during academic year 2019/2020. The total number of FMUI undergraduate medical students from years 1–6 was 1379 students.

### Instrument

Three questionnaires were administered in this study: the Connor-Davidson Resilience Scale (CD-RISC) measured students’ resilience levels, the Brief COPE assessed coping strategies, and the Big Five Personality Inventory examined students’ personality traits.

The Connor-Davidson Resilience Scale (CD-RISC) was developed by Kathryn M. Connor and Jonathan R.T. Davidson to measure resilience among Post-Traumatic Stress Disorder (PTSD) patients. The CD-RISC inventory that we selected is the 25-point inventory with a 0–4 scale. A higher score reflects a higher level of resilience [19]. Brief COPE is a 14-subscale inventory with two items for each subscale, developed by Charles S. Carver for assessing coping strategies. It is a shortened version of the previously developed Cope Inventory. The Brief COPE inventory was used to elucidate the coping mechanisms generally used by respondents. Brief COPE has a Cronbach’s alpha of 0.68–0.90 [20]. The Big Five Personality is an inventory developed through a lexical approach; it attempts to categorize five dimensions of personality: extraversion, agreeableness, conscientiousness, neuroticism, and openness. To measure the level of each personality dimension for this study, respondents were asked to complete a 50-item questionnaire with 1–5 Likert scales [21].

Prior to their use in this study, both the CD-RISC and the Brief COPE inventories were translated from English to Bahasa Indonesia and back-translated to ensure consistency. Following back-translation, we compared the final Bahasa Indonesia versions of the questionnaires to the original versions to ensure their comparable meaning. The Big Five Personality questionnaire used in this study had previously been validated in an Indonesian setting [22].

In addition to questionnaires, we collected grade point average (GPA) data on respondents’ academic achievements from the study program administrators; the maximum GPA is 4.00.

### Data collection

The questionnaires were distributed online through Google Forms for the preclinical students, while clinical students received hard copies from department administrators during their clinical rotations. All respondents provided informed consent to participate in this study.

### Data analysis

The data were analyzed using SPSS version 21. We assessed the reliability of all instruments. An alpha value higher than 0.9 indicates excellent internal consistency; a value of at least 0.7 indicates acceptable internal consistency [23]. The descriptive summary statistics (mean, proportion) were conducted. Descriptive analysis for CD-RISC was conducted by adding together all scores, with a higher score indicating a higher level of resilience (scores range from 0 to 100). The Brief COPE analysis was conducted by summing the subscales and regrouping them into categories of adaptive coping mechanisms: problem-focused (score of 6–24), emotion-focused (score of 10–40), and maladaptive coping mechanisms (score of 12–48) [24, 25]. We analyzed the Big Five Personality test by adding the scores separately for each personality dimension: extraversion (score 8–40), agreeableness (score 9–45), conscientiousness (score 9–45), neuroticism (score 8–40), and openness (score 10–50) [19, 22]. We performed an ANNOVA test to determine differences in the resilience, coping mechanisms, and personality scores of students in each year. Following the descriptive analysis, we conducted post-hoc analysis using the Tukey technique or the Honest Significant Difference (HSD) test to further analyze differences between student groups.

Using Pearson’s analysis, we performed correlation analyses between resilience and each variable (coping mechanisms, the Big Five Personality test, and academic performance). We then analyzed the extent to which independent variables (coping mechanisms, personality, and academic achievement) may explain the variation in resilience scores.

## Results

A total of 1040 respondents completed the questionnaires. The respondents’ characteristics are shown in Table 1.

**Table 1:** Respondents Characteristics

Year	Male	Female	Total Respondents	Total Students	Response Rate (%)
6	53 (40.8)	77 (59.2)	130	222	58.56
5	64 (40.3)	95 (59.7)	159	222	71.62
4	53 (43.1)	70 (56.9)	123	221	55.66
3	93 (48.4)	99 (51.6)	192	232	82.76
2	51 (28.5)	128 (71.5)	179	234	76.50
1	85 (36.0)	151 (64.0)	236	248	95.16
**Grand Total**	**359**	**660**	**1040**	**1379**	**75.42**
Percentage	34.52	63.46	100	–	–

The internal consistency (as measured by Cronbach’s alpha) of the CD-RISC and Brief COPE questionnaires used in this study are very good (Cronbach’s alpha scores of 0.886 and 0.760, respectively), whereas the Big Five Personality test has a Cronbach’s alpha of 0.681. Furthermore, according to the Kolmogorov-Smirnov normality test that we conducted for all subscales in all three questionnaires, the data from the results were normally distributed; therefore, all data in the results are described as means with standard deviations.

FMUI students in both preclinical and clinical stages had moderately high levels of resilience (Table 2), ranging from 64.76 ± 10.69 to 67.60 ± 11.60. Table 2 also shows the coping mechanisms used by students, as well as their GPAs and their personality types according to the Big Five Personality Test. The results suggest that the students had higher scores on adaptive coping (emotion-focused) than on maladaptive coping. Year-1 students had the highest scores; year-5 students had the lowest scores on extraversion, agreeableness, conscientiousness, and openness to experience. This also aligns with GPA scores: year-1 students had higher GPAs than other groups.

**Table 2:** Resilience, Coping Mechanisms, Personality Type, and GPA of Students (*N* = 1040)

Year	Resilience		Coping Mechanisms						Personality										GPA	
	Mean ± SD	P	Problem- focused Mean ± SD	p	Emotion- focused Mean ± SD	p	Mal-adaptive Mean ± SD	p	Extra-version Mean ± SD	p	Agree-ableness Mean ± SD	p	Conscien-tiousness Mean ± SD	p	Neuro-ticism Mean ± SD	p	Open-ness Mean ± SD	p	Mean ± SD	p
6	67.60 ± 11.60	.379	18.23 ± 2.88	.015^a^	29.40 ± 3.84	.268	26.05 ± 4.20	.069	26.92 ± 2.83	.052	28.48 ± 2.96	.024	30.61 ± 2.88	.0001^a^	26.76 ± 2.98	.082	35.51 ± 3.86	.035^a^	3,51 ± 0,17	.0001^a^
5	65.97 ± 11.53		17.88 ± 2.69		28.88 ± 4.28		25.34 ± 3.83		26.29 ± 2.78		27.83 ± 3.02		30.19 ± 2.88		26.34 ± 2.74		34.31 ± 4.03		3,53 ± 0,17	
4	64.76 ± 10.69		17.59 ± 2.46		29.00 ± 4.09		26.43 ± 4.20		26.77 ± 2.50		28.60 ± 2.72		30.25 ± 2.81		26.68 ± 2.77		34.95 ± 3.95		3,44 ± 0,19	
3	65.52 ± 9.80		17.61 ± 2.64		28.90 ± 3.53		26.02 ± 4.46		27.02 ± 2.42		28.71 ± 2.93		31.03 ± 2.78		26.64 ± 2.82		34.67 ± 3.63		3,48 ± 0,22	
2	66.34 ± 11.58		18.42 ± 2.65		29.72 ± 4.07		25.88 ± 3.7		26.79 ± 2.50		28.77 ± 2.95		31.31 ± 2.88		26.98 ± 2.84		34.82 ± 3.71		3,55 ± 0,20	
1	66.64 ± 11.33		18.22 ± 2.71		29.52 ± 3.92		26.63 ± 4.03		27.22 ± 2.87		28.93 ± 3.17		31.23 ± 3.25		27.19 ± 2.83		35.45 ± 3.99		3,75 ± 0,20	

^a^Post-hoc analysis p is significant for:

- Problem-focused between year 3 and year 2. (*p* = 0.040).

- Conscientiousness between year 5 and year 1 (*p =* 0.007) and year 2 (*p* = 0.007), and between year 4 and year 1 (*p* = 0.033) and year 2 (*p* = 0.027).

- Openness between year 5 and year 1 (*p* = 0.047).

- Academic achievement between years 6, 5, 4, 3, 2, and year 1 (*p* = 0.000), and between year 4 and year 2 (*p* = 0.033).

The correlation between personality and resilience was weak-to-moderate for extraversion, agreeableness, conscientiousness, and openness; there was no correlation between neuroticism and resilience. The correlation between coping mechanisms and resilience mostly showed moderate correlation. Students’ GPAs across groups show a very low, yet statistically significant, correlation with resilience. (Table 3).

**Table 3:** Correlation between Resilience, Coping Mechanism, Personality Traits, and Academic Achievement

Year	N	Coping Mechanism								Personality Traits										Academic Achievement	
		Ad	*p*-value	Pr	*p-*value	Em	*p-*value	Mal	*p-*value	Ex	*p-*value	Ag	*p-*value	Cons	*p-*value	Neu	*p-*value	Op	*p-*value	GPA	*p-*value
6	130	0.594	**0.0001**	0.490	**0.0001**	0.576	**0.0001**	0.029	0.740	0.432	**0.0001**	0.272	**0.002**	0.348	**0.0001**	0.026	0.886	0.442	**0.0001**	0.255	**0.003**
5	159	0.647	**0.0001**	0.558	**0.0001**	0.606	**0.0001**	0.205	**0.009**	0.368	**0.0001**	0.248	**0.002**	0.325	**0.0001**	0.070	0.383	0.330	**0.0001**	0.075	0.345
4	123	0.567	**0.0001**	0.502	**0.0001**	0.520	**0.0001**	0.202	**0.025**	0.289	**0.001**	0.155	**0.086**	0.408	**0.0001**	0.073	0.422	0.418	**0.0001**	0.104	0.251
3	192	0.613	**0.0001**	0.524	**0.0001**	0.586	**0.0001**	0.133	0.066	0.309	**0.0001**	0.179	**0.013**	0.187	**0.010**	0.078	0.284	0.271	**0.0001**	0.145	**0.045**
2	179	0.702	**0.0001**	0.659	**0.0001**	0.636	**0.0001**	0.004	0.953	0.383	**0.0001**	0.196	**0.009**	0.265	**0.0001**	0.012	0.869	0.189	**0.011**	0.106	0.158
1	236	0.555	**0.0001**	0.531	**0.0001**	0.477	**0.0001**	0.034	0.604	0.331	**0.0001**	0.285	**0.0001**	0.351	**0.0001**	0.106	0.103	0.430	**0.0001**	0.019	0.770

*Ad* Adaptive coping, *Pr* Problem-focused, *Em* Emotion-focused, *Mal* Maladaptive coping, *Ex* Extraversion, *Ag* Agreeableness, *Cons* Conscientiousness, *Neu* Neuroticism, *Op* Openness

To predict students’ resilience, we completed a regression analysis. The summary of the results is reported in Table 4. Overall, adaptive and maladaptive coping mechanisms, Big Five Personality traits (extraversion, agreeableness, conscientiousness, neuroticism, and openness), and GPA explained 46.9% of students’ resilience.

**Table 4:** Regression coefficients among variables

Variables	Coefficient	t	***p-***value	R^**2**^	F
Constant	− 4.125			**.469**	113.905
Adaptive Score	0.960	21.065	0.0001		
Maladaptive Score	−0.459	−7.119	0.0001		
GPA	0.348	.318	0.751		
Extraversion	0.467	4.264	0.0001		
Agreeableness	0.340	3.408	0.001		
Conscientiousness	0.522	5.174	0.0001		
Neuroticism	−0.476	−4.846	0.0001		
Openness	0.288	3.808	0.0001		

The regression equation in this study is as follows:

$$ \mathbf{Resilience}\ \left(\mathbf{CD}\ \mathbf{RISC}\right)=-\mathbf{4.125}+\mathbf{0.960}\ \mathbf{Adaptive}\ \mathbf{Score}-\mathbf{0.459}\ \mathbf{Maladaptive}\ \mathbf{Score}+\mathbf{0.348}\ \mathbf{GPA}+\mathbf{0.467}\ \mathbf{Extraversion}+\mathbf{0.340}\ \mathbf{Agreeableness}+\mathbf{0.522}\ \mathbf{Conscientiousness}-\mathbf{0.476}\ \mathbf{Neuroticism}+\mathbf{0.288}\ \mathbf{Openness} $$

The value of R Square (0.469) indicates that the effect of the independent variables (adaptive coping score, maladaptive coping score, GPA, extraversion, agreeableness, conscientiousness, neuroticism, and openness) on resilience (CD RISC) is 46.9%. The remaining 53.1% is determined by other variables not considered in study.

According to Table 5, the model indicates that Brief COPE (Adaptive and Maladaptive), GPA, Big Five Personality traits (extraversion, agreeableness, conscientiousness, neuroticism, and openness) together explained 43.3–56.2% of resilience in year 1–6 students.

**Table 5:** Regression analysis of factors contributing to resilience, by year

Year	Β	F	R^**2**^
6	− 27.510	13.732	0,476
5	−12.035	21.565	0.535
4	20.321	18.280	0.562
3	−5.747	22.624	0.497
2	−12.754	24.256	0.533
1	9.507	21.712	0.433

## Discussion

This study aims to identify the level of resilience among undergraduate medical students and its relationships with individual factors: personality, coping mechanisms, and academic performance. We found that the CD-RISC mean score ranged from 64.76 to 67.60. The scores were higher than those from studies among medical students conducted in China (mean score 61.7) or Iran (mean score 62.11) [17, 18]. Studies using CD-RISC in an Asian setting among university students also found a mean score of 60–70 [26–28]. In other settings, such as the USA, UK, and Australia, the range is 70–90 [29–31]. According to Connor and Davidson [19], a higher CD-RISC score indicates greater resilience. Our results fall within quartile 1 of the CD-RISC score (0–73), similar to other studies among medical students in an Asian setting. Culture and sociocultural context are believed to influence coping mechanism and resilience [32, 33] through dynamic interactions of personal traits, cultural values, cultural background and facilitating factors from the environment [32]. Therefore, current results of CD-RISC in Asian setting with hierarchical, collectivist, masculine and uncertainty avoidance cultural context [34] might reflect the dynamic interactions of both personal and environmental factors which influence students’ resilience. For example, expressing individual mental vulnerability might be discouraged in this culture due to competitiveness [35], and high intolerance in dealing with uncertainties coming from the learning environment [36] may progress to individual psychological problems which may reduce the resilience. In addition, the resilience as measured by CD-RISC comes from Western conceptual framework, hence the overall construct might not be transferable in non-Western setting. This calls for further study on understanding resilience concept from multicultural perspectives, especially in non-Western setting [37].

We found that the mean resilience score of year-1 students (66.64) was higher than that of students in years 2–4 (66.34, 65.52, and 64.76, respectively). The mean resilience scores of students in years 5 and 6 seemed to rebound (65.97 and 67.60, respectively). Nevertheless, the mean score difference was not statistically significant; due to the cross-sectional design, we could not report the score change for individual cohorts. Regarding the curriculum, year-4 students were transitioning between preclinical and clinical years, which necessitates adaptation during this period [38] and may entail difficult clinical events [8]. Studies on empathy among undergraduate medical students suggest a similar pattern due to the challenges faced during this transition time [39, 40]. However, the ‘bounce-back’ phenomenon for empathy has been reported in studies in Asia, suggesting the importance of a supportive system and adaptation in a clinical environment [41, 42].

Medical students in years 1–6 showed relatively similar patterns of Big Five Personality Traits. Compared to students from other year levels, Year-1 students showed a greater propensity for fulfilment from external sources (extraversion), more attempts to adjust their behavior to suit others (agreeableness), a high tendency to follow rules (conscientiousness), and a greater willingness to seek new experiences (openness). There were also consistent positive weak-to-moderate correlations between some personality types (extraversion, agreeableness, conscientiousness, and openness) and resilience in medical students across years 1–6. On the other hand, neuroticism did not correlate significantly with resilience. This result aligns with a meta-analysis on the relationship between Big Five personality traits and resilience [43]. The Big Five Personality Test incorporates personality and social cognitive theories [44], which helps explain why self-efficacy is essential for explaining the positive correlation between resilience and the personality traits extraversion, agreeableness, conscientiousness, and openness. Extraversion fosters positive reactions from others [45]; agreeableness helps people initiate new activities and achieve expertise [46]; conscientiousness increases task engagement and effort [47]; openness helps redefine demands as challenges to be overcome [48], which heightens self-efficacy in overcoming adversity. Conversely, neuroticism increases anxiety, which reduces self-efficacy [49].

Coping mechanism, defined as individual’s cognitive and behavioural attempts to deal with demand that are perceived as stressful [50], mostly showed moderate correlations with resilience in our study. Similar results were shown in studies conducted among medical students in South Africa [51], China [16] and United States [3]. Based on the well-being model [52], students’ internal factors, such as personality traits, temperament, and coping styles - acts as a reservoir in dealing with stressors. Students’ choice of coping styles would later affect the resulting condition of being in a state of burnout or becoming a resilient character. The optimal conditions to develop resilience take place when stressors are adequate to be considered as challenges [53]. Moreover, students from all years were found to have higher scores for adaptive coping (emotion-focused scores of 28.88 ± 4.28 to 29.72 ± 4.07; problem-focused scores of 17.59 ± 2.46 to 18.42 ± 2.65) than for maladaptive coping (scores of 25.34 ± 3.83 to 26.63 ± 4.03). Interestingly, students were found to associate more with the emotion-focused coping approach than the problem-focused coping approach. This result is similar to that of a study in South Africa, in which the majority of coping mechanisms chosen by medical students consisted of emotion-focused styles such as positive reframing and religious coping [51]. A study from Germany also found emotion-focused functional coping strategies among their students, such as exercising and seeking support from friends and family [50]. The study by Thompson et al. also suggests that social support plays a major role in the coping mechanisms of medical students [3]. Emotional social support arising from human connection is instrumental in preserving positive coping mechanisms and students’ wellbeing [54]. Moreover, the apparent improvement in coping mechanisms shown by fourth- and fifth-year medical students may result from these cohorts’ increased social interaction as they enter clinical rotations.

Furthermore, a weak correlation was observed between academic performance (GPA) and resilience. We found that GPA was one personal characteristic that showed a very low correlation with resilience across student groups. Resilience can be seen as resulting from the successful interplay of personal and social factors in overcoming challenges, including challenges in medical study [52]. Academic performance may correlate with resilience through academic satisfaction and self-efficacy, which increase self-assuredness [11, 12]. This supports previous findings, which show statistically significant and positive correlations between academic self-efficacy and academic resilience [55]. Self-efficacy is also a significant predictor of academic performance (as measured by GPA) in higher education settings, although not necessarily in undergraduate medical education settings [56]. We did not assess students self-efficacy or academic satisfaction. We also could not assess the dynamics between students’ resilience and their academic performance throughout the year, due to the limitations of our study design. Future studies should account for factors that might influence the correlation between resilience and academic performance, due to the multi-layered and complex nature of resilience.

The regression analysis in this study indicates that 46.9% of resilience could be explained by coping mechanisms, personality, and academic achievement; these personal factors affect resilience variably across year levels (43.4–56.2%). Our study finds that the year-4 students seemed to utilize their personal factors to foster resilience better than students at other year levels. Resilience itself stems from interaction between individuals (including their values, personal characteristics, and prior experiences) and external factors (including social support, learning environment, and cultural and ethical influences) [57, 58]. The dynamics of personal factors that predict resilience among students in our study seem to align with studies in other settings that have separately explored correlations between specific personal characteristics and resilience. Therefore, our study further illuminates the interplay between three personal factors (personality, coping mechanisms, and academic performance) for medical students’ resilience. The adaptive coping mechanisms in our study showed the highest impact on medical students’ resilience. An important aspect of adaptive coping is the use of instrumental and emotional support. Given that Indonesia has a highly collectivist culture, support from peers, family, and teams are generally always available [34]. One study reported that team support enhanced individual coping skills during stressful events and relieved emotional hardship; thus, it may also improve individual resilience [59].

Our study showed constant level of resilience across study years, which was affected by personal factors. However, it was known that resilience can be learned and improved overtime through a systematic intervention [14, 60]. Therefore, the results emphasized the importance of facilitating students with periodic self-assessment of their coping mechanism, personality, academic achievement as well as significant experiences, while actively reflecting on those aspects to know themselves better. Such opportunities may help them to identify whether they are in needs for further support from peers or faculty members, hence increasing their resilience overtime. Our findings also strongly support efforts to provide personalized learning for medical students in the current era [61]. Identifying personal factors that impact resilience among medical students enables students to optimize their potential to overcome challenges in their studies. This also enables medical teachers and medical schools to provide necessary support for medical students, considering both personal factors and external challenges (e.g., curriculum, assessment, learning environment). It is equally important to further explore the external factors that affect resilience in this setting to further support medical students’ resilience and wellbeing [62].

This study has several limitations. First, due to the limitations of a cross-sectional design, our results cannot explain the causal relationships between resilience and the personal factors being measured. Nevertheless, we have elaborated the dynamics between some personal factors and their correlations with resilience among medical students. Second, the personal factors we measured only partially predict medical students’ resilience. Further research is needed to elucidate the relationship between medical students’ resilience and personal factors such as self-efficacy or external factors such as social support and the learning environment.

## Conclusion

The levels of resilience among undergraduate medical students were similar across years 1–6; these levels were comparable to resilience scores among undergraduate medical students in other settings, especially in Asia. Our results indicate that coping mechanisms, personality traits (according to the Big Five Personality Test), and academic performance may predict resilience. The adaptive coping mechanism seems to be highly important for cultivating resilience among medical students. This study strongly suggests the importance of identifying personal factors that support resilience in this population, as part of efforts to provide students with support and personalized learning.

## Data Availability

The datasets generated and/or analysed during the current study are not publicly available due to conditions of participants’ consents but are available from the corresponding author on reasonable request.
